# Fast and Accurate Electric Field Gradient Calculations in Molecular Solids With Density Functional Theory

**DOI:** 10.3389/fchem.2021.751711

**Published:** 2021-10-07

**Authors:** Joshua D. Hartman, Amanda Mathews, James K. Harper

**Affiliations:** ^1^ Department of Chemistry, Mt. San Jacinto College, Menifee, CA, United States; ^2^ Department of Chemistry and Biochemistry, Brigham Young University, Provo, UT, United States

**Keywords:** nuclear magnetic resonance, electric field gradient, fragment methods, GIPAW, crystal structure, nuclear quadrupole coupling, 17-O

## Abstract

Modern approaches for calculating electric field gradient (EFF) tensors in molecular solids rely upon plane-wave calculations employing periodic boundary conditions (PBC). In practice, models employing PBCs are limited to generalized gradient approximation (GGA) density functionals. Hybrid density functionals applied in the context of gauge-including atomic orbital (GIAO) calculations have been shown to substantially improve the accuracy of predicted NMR parameters. Here we propose an efficient method that effectively combines the benefits of both periodic calculations and single-molecule techniques for predicting electric field gradient tensors in molecular solids. Periodic calculations using plane-wave basis sets were used to model the crystalline environment. We then introduce a molecular correction to the periodic result obtained from a single-molecule calculation performed with a hybrid density functional. Single-molecule calculations performed using hybrid density functionals were found to significantly improve the agreement of predicted ^17^O quadrupolar coupling constants (*C*_*q*_) with experiment. We demonstrate a 31% reduction in the RMS error for the predicted ^17^O *C*_*q*_ values relative to standard plane-wave methods using a carefully constructed test set comprised of 22 oxygen-containing molecular crystals. We show comparable improvements in accuracy using five different hybrid density functionals and find predicted *C*_*q*_ values to be relatively insensitive to the choice of basis set used in the single molecule calculation. Finally, the utility of high-accuracy ^17^O *C*_*q*_ predictions is demonstrated by examining the disordered 4-Nitrobenzaldehyde crystal structure.

## 1 Introduction

Solid-state nuclear magnetic resonance (SSNMR) spectroscopy has proven highly effective at characterizing molecular crystals. Advances in NMR hardware and the development of novel pulse sequences continue to improve the accuracy and availability of experimental data. However, mapping relationships between NMR observables and structural features remains a formidable challenge. SSNMR investigations are often coupled with X-ray diffraction and first-principals calculations to form the interdisciplinary field of NMR crystallography. The success of NMR crystallography has been greatly facilitated by the availability of accurate computational methods for predicting NMR parameters which typically employ density functional theory (DFT) (Gervais et al., 2005; Wu, 2008; Kong et al., 2013; Yang et al., 2016; Kong et al., 2017; Soss et al., 2017; Holmes and Schurko, 2018; Yamada et al., 2020; Chalek et al., 2021; Wang et al., 2021).

There are two broad classifications for DFT-based methods commonly applied to molecular crystals. First, the gauge-including projected augmented wave (GIPAW) method (Pickard and Mauri, 2001) employs plane-wave basis sets to naturally capture the periodic nature of the crystalline lattice. Alternatively, the gauge-including atomic orbital (GIAO) approach (Ditchfield, 1974; Wolinski et al., 1990) relies on fragments or clusters of molecules constructed to mimic the crystalline environment (Hartman and Beran, 2014; Holmes et al., 2014; Hartman et al., 2015). Plane-wave and GIAO-based calculations have both proven highly effective in modeling a range of NMR parameters derived from the chemical shielding (CS) tensor and the electric field gradient (EFG) (Nakajima, 2017; Holmes and Schurko, 2018; Dračínský et al., 2019; Gregorovič, 2020).

Plane-wave methods have a natural advantage over fragment and cluster-based calculations when predicting NMR parameters for molecular crystals due to the explicit quantum mechanical treatment of crystal lattice effects. However, plane-wave methods are limited in practice to density functionals based on the generalized gradient approximation (GGA). Numerous studies have shown that using hybrid density functionals improves the accuracy of predicted NMR parameters (Hartman and Beran, 2014; Holmes et al., 2014; Hartman et al., 2015; Hartman et al., 2017). Previous benchmark studies involving ^1^H, ^13^C, ^15^N and ^51^V nuclei have shown fragment methods with hybrid density functionals improve the accuracy of predicted isotropic chemical shifts (Hartman et al., 2016; Hartman et al., 2017; Mathews and Hartman, 2021). Fragment methods employing hybrid density functionals coupled with electrostatic embedding techniques have demonstrated improved accuracy in the prediction of CS tensor principal components (Hartman and Beran, 2018) and predicted *C*_*q*_ values for ^14^N (Gregorovič, 2020).

Recently, a novel approach involving GIPAW with a molecular correction (GIPAW + MC) was put forward for modeling the chemical shift tensor (Nakajima, 2017; Dračínský et al., 2019). This scheme combines the strengths of plane-wave and molecular calculations. The GIPAW + MC approach relies upon GGA-based GIPAW calculations to capture long-range effects and then introduces a correction obtained from a molecular calculation performed on an isolated gas-phase molecule. The geometry of the isolated molecule is taken from the optimized crystal structure. In this way, hybrid density functionals or even post-Hartree-Fock wave function methods can be used in the molecular calculation to more accurately model the intramolecular effects on the CS tensor. A more detailed description of the GIPAW + MC method applied to CS tensor predictions can be found in the literature (Nakajima, 2017; Dračínský et al., 2019; Bártová et al., 2020).

Applying the GIPAW + MC approach (vide infra) to CS tensors has proven particularly effective in modeling NMR parameters for the quadrupolar ^17^O nucleus (Dračínský et al., 2019). Specifically, GIPAW + MC calculations using the PBE0 hybrid density functional and a 6-311+G (2d,p) basis reduces the mean absolute error (MAE) for predicted ^17^O chemical shifts by 17% relative to GIPAW (Dračínský et al., 2019). Improving the accuracy of predicted NMR parameters for quadrupolar nuclei is of particular interest given that quadrupolar nuclei account for approximately 73% of NMR-active nuclei (Hamaed et al., 2010).

In addition to improving the accuracy of CS tensor predictions, monomer correction methods have proven successful in modeling the energetics of conformational polymorphs (Greenwell and Beran, 2020). Here we extend the GIPAW + MC model to EFG tensor calculations and apply GIPAW + MC tensor calculations to a benchmark set of 22 molecular crystals with a total of 46 unique ^17^O environments. We demonstrate a 31% improvement in the accuracy of predicted *C*_*q*_ values relative to traditional plane-wave methods with a negligible increase in computational cost. These findings are particularly promising for NMR crystallography applications given the crucial role EFG tensor predictions have come to play in crystal structure refinement (Holmes and Schurko, 2018; Yamada et al., 2020), understanding hydrogen bond properties (Kazuhiko et al., 2000; Samadi et al., 2008) and investigating chemical reactivity and dynamics (Ashbrook and Sneddon, 2014; Bernard et al., 2018).

In the following, benchmark data are employed to examine basis set convergence and the accuracy of the predicted *C*_*q*_ values for Pople, core-valence Dunning-type basis sets, and the pcs-*n* (*n* = 1–4) series of basis sets. The performance of six commonly used density functionals are compared to demonstrate uniform improvement in accuracy through the application of a range of hybrid density functionals in the molecular calculation. We examine the relative improvement in EFG and CS tensor GIPAW calculations through the application of a molecular correction. Finally, GIPAW + MC EFG calculations are shown to accurately predict *C*_*q*_ values for the disordered oxygen atoms in the 4-Nitrobenzaldehyde crystal structure.

## 2 Theory and Methods

The EFG tensor is obtained from the second spatial derivative of the electrostatic potential *V* resulting from the charge distribution surrounding the nucleus.

$$V_{\alpha \beta }=\frac{\partial^{2}V}{\partial x_{\alpha }\partial x_{\beta }}.$$ (1)

Following Eq. 1, the EFG tensor is a symmetric 3 × 3 tensor with zero trace. Diagonalization of the EFG tensor yields three principal components defined such that |*V*_33_|≥|*V*_22_|≥|*V*_11_|. The principal components of the EFG tensor are used to derive two NMR observables. First, the nuclear quadrupolar coupling constant *C*_*q*_ is obtained from *V*_33_ according to,

$$C_{q}=eQV_{33}/h$$ (2)

where *e* is the elementary charge, *Q* = −25.58 *mb* (Pyykkö, 2001) is the nuclear quadrupole moment for ^17^O, and *h* is Plank’s constant. Second, the asymmetry parameter *η*_*q*_ is obtained from the ratio of the difference in *V*_11_ and *V*_22_ to *V*_33_.

$$\eta_{q}=\left(V_{11}-V_{22}\right)/V_{33}$$ (3)

Minor structural changes can have a pronounced impact on both *C*_*q*_ and *η*_*q*_. However, previous studies suggest that the impact of structural changes on the EFG tensor is highly local (Gregorovič, 2020), and the GIPAW + MC approach to EFG tensor calculations is motivated by this assumption. The GIPAW + MC EFG tensor (*V*_*corr*_) is constructed from three separate calculations as follows,

$$V_{corr}=V_{cryst}^{GIPAW}-V_{mol}^{low}+V_{mol}^{high}.$$ (4)

First, the EFG tensor is computed using a full plane-wave GIPAW calculation at the lower level of theory (typically PBE) to obtain $V_{cryst}^{GIPAW}$ . Individual molecule calculations are then carried out at the lower level of theory to obtain $V_{mol}^{low}$ and the higher level of theory to obtain $V_{mol}^{high}$ . Both molecule calculations are performed using coordinates obtained after optimization in the plane-wave environment. The corrected EFG tensor is then computed according to Eq. 4.

According to Eq. 4, the GIPAW + MC model treats intermolecular interactions within the crystalline lattice using plane-wave methods. Critical intramolecular effects are then included in the form of a molecular correction. Molecular calculations performed within the GIAO framework can readily accommodate a higher level of theory since both *V*_*mol*_ terms rely on isolated gas-phase molecule calculations. Finally, the corrected EFG tensor is then subject to diagonalization to obtain the principal components which are used along with Eqs 2, 3 to predict the NMR observables.

## 3 Computational Methods

Molecular crystals were selected for inclusion in the benchmark study based on the availability of high-quality X-ray diffraction data and experimental NMR data providing high-accuracy *C*_*q*_ values (see Table 1). In all cases, both the experimental diffraction and NMR data were obtained at room temperature. Structural data from diffraction studies are obtained from the Cambridge Structure Database (CSD) maintained by the Cambridge Crystallographic Data Center. The CSD reference codes and the experimental references for NMR data are provided in Table 2. The crystal structure for 4-Nitrobenzaldehyde (KAYSUY) with a disordered oxygen is used as an application (Wu et al., 2008).

**TABLE 1:** Experimental^17^O *C*_*q*_ values with the reported uncertainty for each structure in the benchmark set. Calculated *C*_*q*_ for GIPAW and GIPAW + MC calculations provided along with the absolute errors. All values are reported in MHz and the GIPAW + MC calculations were performed with a PBE0/cc-pCVTZ correction.

CSD code				GIPAW		GIPAW + MC	
	Atom	‖*Exp*.‖	Uncer.	‖*Calc*.‖	Abs. Error	‖*Calc*.‖	Abs. Error
TAURIN01	O1	6.70	0.03	7.56	0.86	7.17	0.47
	O2	6.65	0.03	7.36	0.71	7.00	0.35
	O3	6.80	0.03	7.49	0.69	7.11	0.31
BZANIL02	O1	8.97	0.02	9.30	0.33	8.90	0.07
TPEPHO02	O1	4.57	0.01	5.18	0.61	4.99	0.42
PHALNC01	O1	8.55	0.08	8.93	0.38	8.47	0.08
	O2	7.41	0.08	7.66	0.25	7.38	0.03
VALEHC11	O1	8.41	0.08	8.88	0.47	8.41	0.00
	O2	7.35	0.08	7.67	0.32	7.40	0.05
GLUTAM01	O1	8.10	0.05	8.54	0.44	7.99	0.11
	O2	7.25	0.05	7.50	0.25	6.98	0.27
LHISTD13	O1	7.35	0.05	7.74	0.39	7.20	0.15
	O2	7.50	0.05	8.01	0.51	7.65	0.15
THYMIN01	O1	6.65	0.05	7.30	0.65	7.04	0.39
	O2	8.40	0.05	9.13	0.73	8.76	0.36
CUWKIO	O1	7.90	0.08	7.93	0.03	7.51	0.39
	O2	7.05	0.08	6.94	0.11	6.74	0.31
URACIL	O1	7.61	0.05	8.21	0.60	7.89	0.28
	O2	7.85	0.05	7.53	0.32	7.20	0.65
MOHCIW	O1	8.35	0.08	8.92	0.57	8.48	0.13
	O2	7.60	0.08	7.77	0.17	7.54	0.06
LALNIN12	O1	7.80	0.05	8.60	0.80	7.99	0.19
	O2	6.70	0.05	6.80	0.10	6.25	0.45
ALAHCL	O1	8.30	0.03	8.71	0.41	8.24	0.06
	O2	7.30	0.04	7.60	0.30	7.33	0.03
MBNZAM10	O1	8.50	0.02	8.89	0.39	8.52	0.02
ACANIL03	O1	8.81	0.02	9.24	0.43	8.90	0.09
LTHREO01	O1	7.40	0.09	7.89	0.49	7.33	0.07
	O2	7.30	0.09	7.89	0.59	7.44	0.14
LTYROS11	O1	7.50	0.05	7.86	0.36	7.29	0.21
	O2	6.70	0.05	6.63	0.07	6.12	0.58
LTYRHC10	O1	8.22	0.05	8.58	0.36	8.28	0.06
	O2	7.35	0.05	7.45	0.10	7.14	0.21
	O3	8.56	0.05	8.77	0.21	8.46	0.10
SALIAC12	O1	7.40	0.05	7.72	0.32	7.51	0.11
	O2	7.10	0.05	6.68	0.42	6.22	0.88
	O3	8.30	0.05	8.39	0.09	8.02	0.28
ACSALA17	O1	6.50	0.05	7.69	1.19	7.27	0.77
	O2	6.60	0.05	7.47	0.87	7.25	0.65
	O3	9.50	0.05	9.99	0.49	9.63	0.13
	O4	8.70	0.05	9.20	0.50	8.79	0.09
TICHOC	O1	8.10	0.05	8.15	0.05	7.79	0.31
	O2	8.00	0.05	7.93	0.07	7.49	0.51
	O3	9.20	0.05	9.68	0.48	9.45	0.25
LASPRT	O1	7.60	0.05	8.01	0.41	7.45	0.15
	O2	6.90	0.05	7.00	0.10	6.44	0.46
RMSE (MHz)					0.48		0.33
max abs. Error					1.19		0.88

**TABLE 2:** Chemical name, CSD reference code, and experimental NMR reference for all crystal structures included in the^17^O benchmark study.

Name	Ref. Code	Exp. NMR ref.
*β*-Alanine sulfonic acid	TAURIN01	Kong et al. (2012)
N-phenylbenzamide	BZANIL02	Kazuhiko et al. (2000)
Triphenylphosphine oxide	TPEPHO02	Bryce et al. (2003)
L-Phenylalanine hydrochloride	PHALNC01	Pike et al. (2004)
L-Valine hydrochloride	VALEHC11	Gervais et al. (2005)
L-Glutamine	GLUTAM01	Yamada et al. (2007a)
L-Histidine	LHISTD13	Yamada et al. (2007a)
Thymine	THYMIN01	Wu et al. (2002)
Fmoc-L-alanine monohydrate	CUWKIO	Yamada et al. (2008a)
Uracil	URACIL	Wu et al. (2002)
Fmoc-N (Me)Ser(tBu)-OH	MOHCIW	Yamada et al. (2008a)
L-Alanine	LALNIN12	Gervais et al. (2005)
L-Alanine hydrochloride	ALAHCL	Yamada et al. (2008b)
N-Methylbenzamide	MBNZAM10	Yamada et al. (2000)
Acetanilide	ACANIL03	Yamada et al. (2000)
L-Threonine	LTHREO01	Yamada et al. (2007b)
L-Tyrosine	LTYROS11	Yamada et al. (2007b)
L-Tyrosine hydrochloride	LTYRHC10	Pike et al. (2004)
2-Hydroxybenzoic acid	SALIAC12	Kong et al. (2013)
2-acetoxybenzoic acid	ACSALA17	Kong et al. (2013)
2-Ethanoylbenzoic acid	TICHOC	Kong et al. (2017)
LASPRT	LASPRT	Yamada et al. (2007b)

### 3.1 Crystal Structure Optimization

The experimental X-ray diffraction structures were used as a starting point for all-atom geometry optimizations subject to fixed experimental room-temperature lattice parameters. All-atom geometry optimization was carried out using dispersion-corrected DFT with the D3 dispersion correction (Grimme et al., 2010) and a maximum *k*-point spacing of 0.05 Å ^−1^. The open-source Quantum Espresso (Giannozzi et al., 2009) software package, the PBE density functional, and an 80 Ry plane-wave cutoff were employed for the geometry optimizations. The following ultrasoft pseudopotentials were used: H.pbe-rrkjus.UPF, C.pbe-rrkjus.UPF, N.pbe-rrkjus.UPF, O.pbe-rrkjus.UPF, S.pbe-n-rrkjus_psl.0.1.UPF, Cl.pbe-n-rrkjus_psl.0.1.UPF, P.pbe-n-rrkjus_psl.0.1.UPF. All pseudopotentials used in the present work can be obtained from http://www.quantum-espresso.org.

### 3.2 EFG Tensor Calculations

Gauge-including projector augmented wave (GIPAW) chemical shielding calculations were performed using the optimized geometries. Calculations were performed using CASTEP (Clark et al., 2005) with the PBE density functional, ultrasoft pseudopotentials generated on-the-fly, and an 850 eV plane-wave basis set cut-off. Sampling for *k*-points was performed on a Monkhorst–Pack grid to give a maximum separation between k-points of 0.05 Å^−1^. These parameters were chosen based on previous benchmark studies involving quadrupolar nuclei (Hartman et al., 2016; Mathews and Hartman, 2021). Full space group symmetry was used in all GIPAW calculations.

EFG tensor calculations for the isolated molecules were carried out using Gaussian09 (Frisch et al., 1984) and the PBE0 (Adamo and Barone, 1999), PBE (Perdew et al., 1996), B3LYP (Stephens et al., 1994), TPSSh (Staroverov et al., 2003), *ω*B97XD (Chai and Head-Gordon, 2008), and mPW1PW91 (Adamo and Barone, 1998) density functionals. A large DFT integration grid consisting of 150 radial and 974 Lebedev angular points was selected on the basis of previous work (Hartman and Beran, 2014). The large integration grid approaches rotational invariance thereby reducing noise in the monomer calculations. To explore basis set dependence three classes of basis sets were employed. The Pople basis set 6-311+G (2d,p) (Frisch et al., 1984; Clark et al., 1983) was used to facilitate direct comparison with previous work (Hartman et al., 2016; Dračínský et al., 2019; Mathews and Hartman, 2021). The Dunning-type core-valance basis sets (Woon and Dunning, 1995), were used to examine the impact of tight higher angular momentum basis functions. Finally, the pcs-*n* (*n* = 1–4) basis sets were used to determine if the accuracy of predicted *C*_*q*_ values could be improved using basis sets optimized for predicting NMR parameters. The Dunning-type and pcs-*n* basis sets where obtained from the basis set exchange (https://bse.pnl.gov/bse/portal) (Pritchard et al., 2019).

## 4 Results and Discussion

### 4.1 ^17^O Quadrupole Coupling Constants

We compare the accuracy of GIPAW + MC predicted ^17^O EFG tensor parameters with those obtained experimentally from NMR and NQR spectroscopy. We have selected the quadrupolar ^17^O nucleus for two reasons. First, ^17^O has tremendous biological and pharmaceutical importance. Second, previous GIPAW + MC studies involving the CS tensor showed the largest magnitude improvement in predicted isotropic shieldings for ^17^O relative to other second-row nuclei (Dračínský et al., 2019). These findings are consistent with previous fragment-based investigations which showed predicted isotropic shieldings for ^17^O to be more sensitive to long-range electrostatic effects relative to hydrogen and nitrogen (Hartman et al., 2016; Hartman et al., 2017).

We have selected 22 crystal structures with 46 unique ^17^O environments to benchmark the accuracy of GIPAW + MC EFG tensor predictions. The experimental *C*_*q*_ values in the benchmark set range from 4.57 to 9.50 MHz with an experimental uncertainty ≤0.09 MHz. Table 1 provides a complete list of all structures included in the benchmark analysis along with the experimental *C*_*q*_ values and the reported uncertainty. The crystal structures with labeled oxygen atoms are depicted in the SI. Table 1 also provides the predicted *C*_*q*_ values for traditional plane-wave calculations (GIPAW) and those obtained from the GIPAW + MC calculations. In most cases, the sign of *C*_*q*_ cannot be determined from the NMR experiment therefore we provide the magnitudes of the predicted *C*_*q*_ values and report the absolute error (in MHz) relative to experiment.

Table 1 establishes improved accuracy in the predicted *C*_*q*_ values obtained from GIPAW + MC calculations relative to traditional GIPAW. Specifically, introducing a molecular correction carried out at the PBE0/cc-pCVTZ level reduces the RMS error by 31% and reduced the maximum absolute error by 26%. The choice of density functional and basis set used in the preliminary analysis was motivated by previous studies (Harbison, 2015; Dračínský et al., 2019). In the following sections we thoroughly examine basis set convergence and the relative performance of different hybrid density functionals.

### 4.2 Basis Set Convergence

Previous studies applying the GIPAW + MC approach to CS tensor calculations on second-row nuclei found the method to be relatively insensitive to the choice of basis set used in the molecular correction (Dračínský et al., 2019). However, the regression model used to compare the absolute shieldings obtained from CS tensor calculations to the experimental chemical shifts partially corrects for systematic error. Unlike the CS tensor, comparing predicted EFG tensors (Eqs 2, 3) with experiment does not involve regression and therefore does not benefit from systematic error correction. In addition, previous studies involving GIAO-based EFG tensor predictions demonstrated improved accuracy in the predicted *C*_*q*_ values through the introduction of tight *d* functions (Harbison, 2015). Therefore, care must be taken to ensure the predicted EFG tensor components are well-converged with respect to basis set.

**FIGURE 1:**
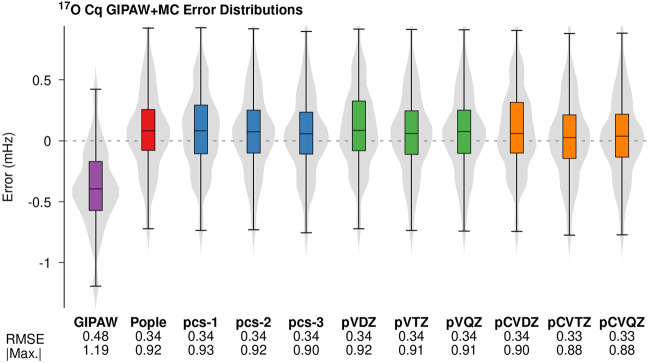
Errors in the ^17^O *C*_*q*_ predictions from PBE/GIPAW (purple) and GIPAW + MC calculations with PBE0 monomer corrections performed using different Gaussian basis sets. The PBE0/Pople basis (red) corresponds to the data in Table 1. The violin plots illustrate kernel density estimates for each error distribution. The box-plots within each violin provide the median (black horizontal line), middle two quartiles (colored box), and the whiskers represent the outer quartiles. The corresponding RMS error and maximum absolute errors are provided below each distribution. All values are reported in MHz.

Figure 1 illustrates the error distributions for the predicted *C*_*q*_ values and the corresponding RMS error for GIPAW + MC calculations employing the PBE0 hybrid density functional in the molecular correction. Figure 1 provides error distributions for all three classes of basis functions and the corresponding data is provided for traditional plane-wave calculations (GIPAW) in purple. The pCVTZ basis results shown in orange correspond to the data presented in Table 1. The ^17^O *C*_*q*_ error distributions, RMSE, and maximum absolute errors yield comparable performance across the different classes of basis sets. The PBE0 molecular corrections provide ∼30% improvement in RMS error relative to GIPAW. In all cases, the GIPAW + MC results are well-converged at the double-*ζ* level.

**FIGURE 2:**
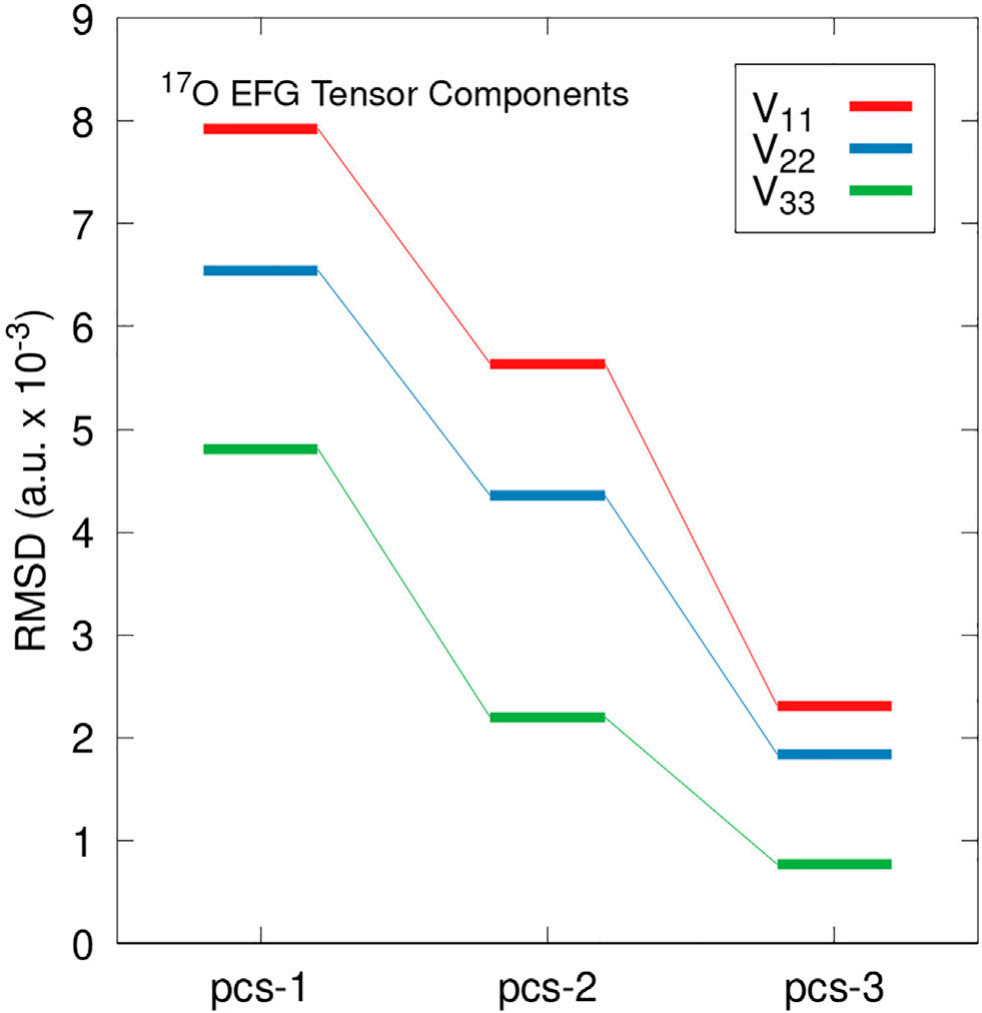
RMS deviations in predicted EFG tensor principal components relative to PBE0/pcs-4 calculations. Deviations in the |*V*_33_| component are shown in red, |*V*_22_| in blue, and |*V*_11_| in green. Values are reported in atomic units with a scaling factor of 10^–3^.

Predicted *C*_*q*_ values rely on the largest magnitude principal component of EFG tensor (*V*_33_). To ensure convergence extends to all principal components we examine the deviation in all three principal components obtained from PBE0/pcs-*n* EFG tensor predictions for *n* = 1–3 relative to pcs-4. Figure 2 illustrates the RMS deviation in |*V*_33_| (red), |*V*_22_| (blue), and |*V*_11_| (green) relative to pcs-4 calculations applied to the benchmark set. The largest RMS deviations were observed for the largest EFG tensor component, *V*_33_, followed by *V*_22_, and *V*_11_ yields the smallest deviation. Together, Figures 1, 2 show EFG tensor predictions to be well-converged using standard triple-*ζ* basis sets. These findings are in agreement with previous work involving Dunning-type basis sets (Wu et al., 2008).

Figures 1, 2 suggest ^17^O *C*_*q*_ GIPAW + MC predictions employing DFT methods are relatively insensitive to the choice of basis set. The Dunning-type core-valance basis sets which include tight higher angular momentum basis functions (orange in Figure 1) do show a slight reduction (∼0.02 MHz) in the maximum absolute error. However, this improvement is equal to the average experimental uncertainty of the benchmark set. This result is surprising given previous results employing wave function-based correlation methods that showed improved accuracy in the predicted *C*_*q*_ values through the introduction of tight *d* functions (Harbison, 2015). Extending the GIPAW + MC model to wave function methods with custom Gaussian basis sets is a topic of an ongoing investigation.

### 4.3 Relative Performance of Density Functionals

The accuracy of predicted NMR parameters have been shown to vary substantially with different density functionals (Holmes et al., 2015). However, comparable performance is often observed within a given class of density functionals (Hartman et al., 2016). The results in the previous section establish the improved accuracy in *C*_*q*_ predictions through the introduction of a molecular correction based on the PBE0 hybrid density functional. In this section, we examine the relative performance of five other commonly used density functionals.

**FIGURE 3:**
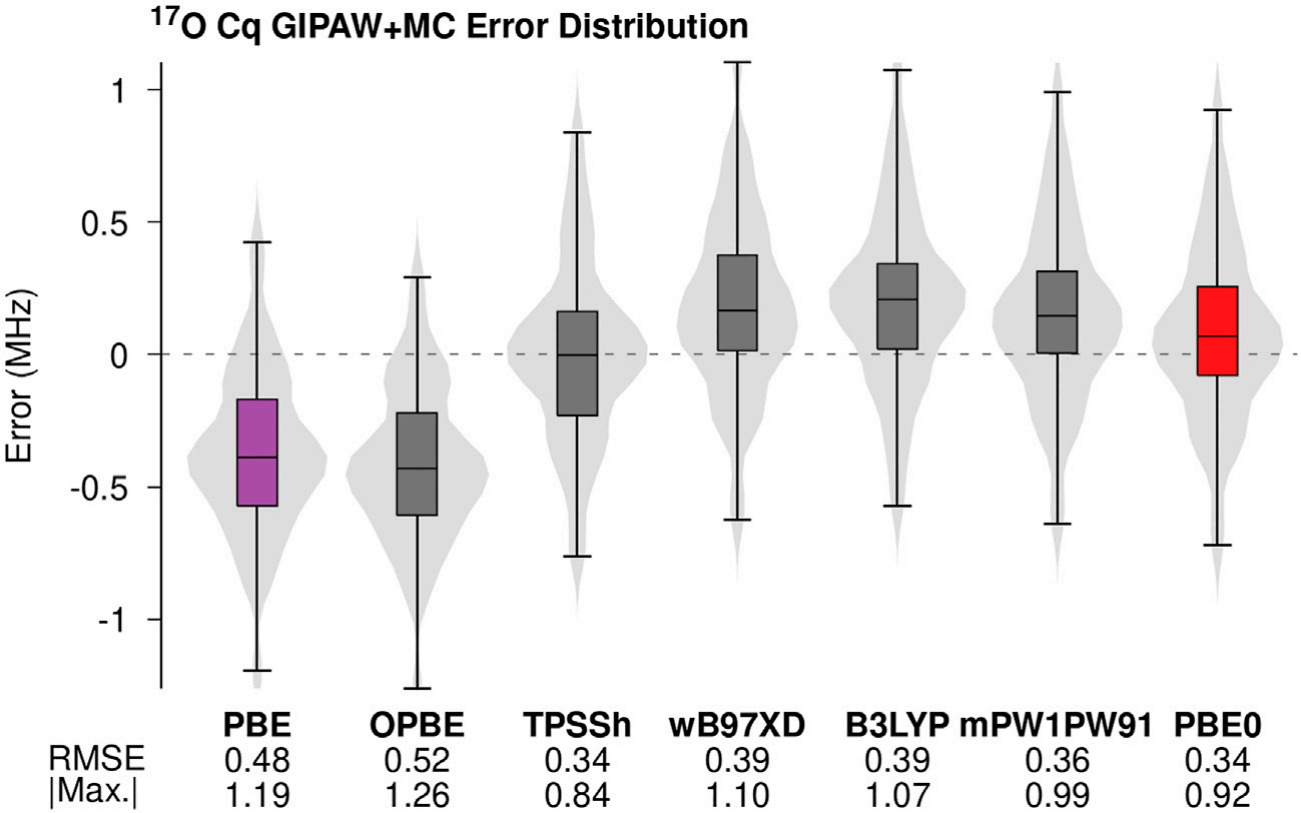
Errors in the predicted ^17^O *C*_*q*_ values for GIPAW and GIPAW + MC calculations were performed using a selection of commonly used density functionals. The molecular corrections were performed using the 6-311+G (2d,p) basis.

Figure 3 provides the error distributions associated with GIPAW + MC ^17^O *C*_*q*_ predictions using six common density functionals. GIPAW/PBE results are included in purple for comparison. A previous study identified the OPBE density functional as the best GGA-based density functional for predicting ^13^C, ^15^N, ^17^O, and ^19^F chemical shifts (Zhang et al., 2006). More recently, a benchmark study applying fragment methods to the prediction of isotropic ^17^O chemical shieldings in molecular crystals showed a slight improvement in the accuracy of OPBE relative to the PBE density functional (Hartman et al., 2016). Comparing the RMSE and maximum absolute error for GIPAW/PBE and GIPAW + MC/OPBE in Figure 3 suggests that the improved performance for OPBE in terms of predicting isotropic chemical shieldings does not translate to ^17^O *C*_*q*_ calculations.

As expected, molecular corrections carried out using hybrid density functionals (TPSSh, wB97XD, mPW1PW91, B3LYP, and PBE0) uniformly improve the accuracy of predicted ^17^O *C*_*q*_ values relative to both GGA-based predictions. Recent work suggests double-hybrid density functionals further improves the accuracy of NMR parameter predictions (Stoychev et al., 2018). Extending the GIPAW + MC analysis to include double-hybrid density functionals is a topic of ongoing investigation. The RMS errors obtained for the different hybrid density functionals agree to within the maximum experimental uncertainty. However, the meta-hybrid TPSSh does show a small improvement in the maximum absolute error relative to the other density functionals. The hybrid density functional PBE0 (red in Figure 3) yields the second-lowest maximum absolute error. Once again, these findings represent a small deviation from previous studies involving ^17^O isotropic shielding calculations in which PBE0 and B3LYP predictions improved the accuracy relative to TPSSh (Hartman et al., 2016). Minor deviations aside, the trends in density functional choice illustrated in Figure 3 support the general consensus that hybrid density functionals improve the accuracy of predicted NMR parameters relative to GGA (Holmes et al., 2014; Holmes et al., 2015; Hartman et al., 2016; Hartman et al., 2017; Hartman and Beran, 2018).

### 4.4 Accuracy of Predicted ^17^O CS and EFG Tensors

Predicted ^17^O NMR parameters are highly sensitive to crystalline lattice effects (Hartman et al., 2016; Hartman et al., 2017). Previous work involving fragment-based methods coupled with electrostatic embedding have shown improved accuracy in the predicted isotropic shieldings for ^1^H, ^13^C, ^15^N, and ^51^V relative to GIPAW/PBE when hybrid density functionals are employed (Hartman et al., 2015; Hartman et al., 2016; Mathews and Hartman, 2021). However, GIPAW/PBE calculations yield more accurate ^17^O isotropic chemical shift predictions compared to fragment models with hybrid density functionals and self-consistent embedding (Hartman et al., 2017). Additionally, comparing predicted isotropic chemical shifts obtained from GIPAW + MC and GIPAW calculations shows a larger improvement in accuracy for ^17^O nuclei relative to both ^13^C and ^15^N (Dračínský et al., 2019). Specifically, GIPAW + MC improves the RMS error by 27% and reduces the maximum error by 26% relative to GIPAW/PBE (Dračínský et al., 2019). There findings suggest faithful reproduction of crystal lattice effects are essential for high-accuracy ^17^O NMR parameter prediction.

Based on the success in predicting ^17^O EFG parameters using GIPAW + MC, this approach was employed to also evaluate the accuracy of GIPAW + MC in predicting ^17^O CSA tensor components. This analysis represents the first benchmark study examining the accuracy of predicted ^17^O CSA tensor components obtained from GIPAW + MC calculations. We compare the accuracy of predicted ^17^O isotropic shieldings and CSA tensor elements with predicted *C*_*q*_ values for both GIPAW and GIPAW + MC. Because all three types of experimental data are not available for each benchmark compound, different numbers of compounds are included in each comparison. Specifically, the *C*_*q*_ error distribution and isotropic shielding data both include all 22 crystal structures in the benchmark set. The CSA tensor data includes 21 structures. To facilitate comparison with previous studies, the molecular correction was performed at the PBE0/6-311+G (2d,p) level and standard linear regression methods were used to map the predicted absolute shieldings to experiment. The details of the ^17^O regression models along with the experimental and calculated CS tensor data are provided in the supporting information.

**FIGURE 4:**
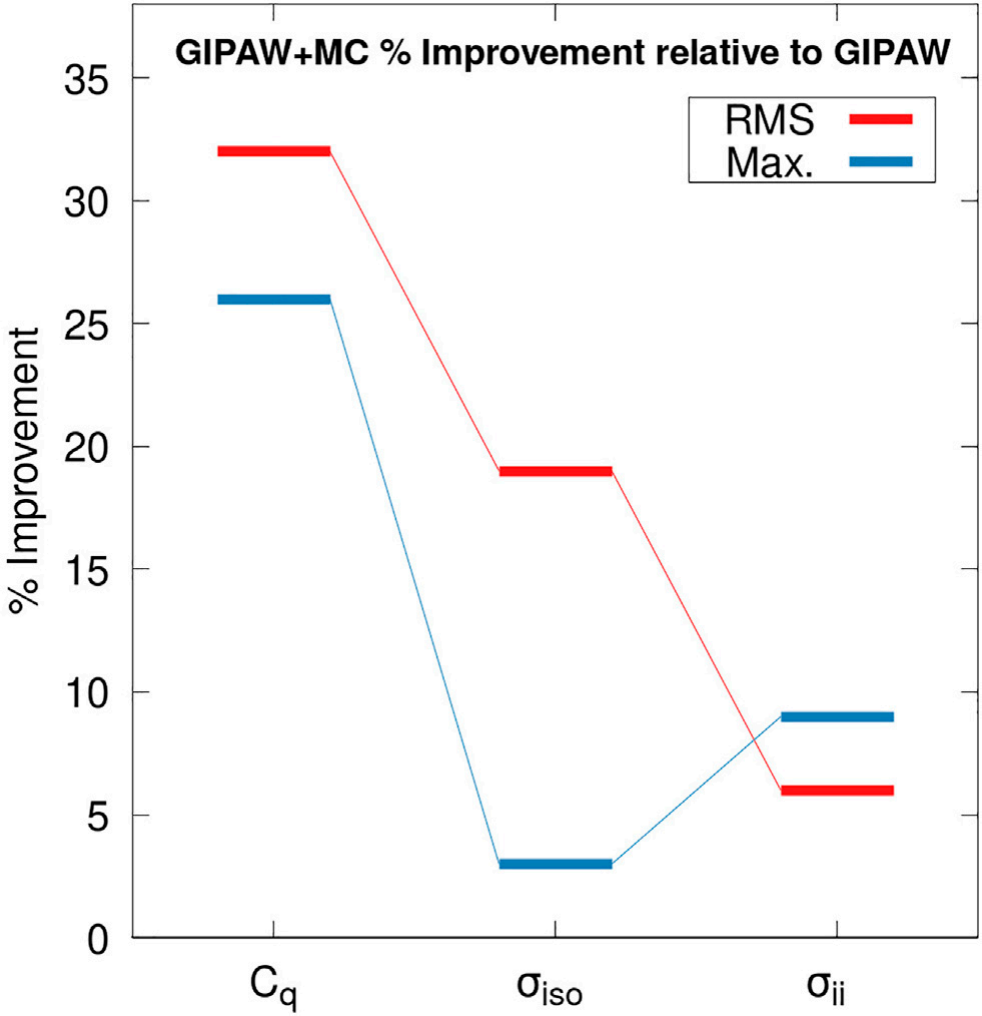
Percent improvement in the RMS error (red) and the maximum absolute error (blue) for GIPAW + MC calculations relative to GIPAW/PBE. Results are reported for the ^17^O quadrupole coupling constant (*C*_*q*_), the isotropic chemical shift (*σ*_*iso*_), and the CSA tensor components (*σ*_*ii*_). The molecular correction for the GIPAW + MC calculations were performed at the PBE0/6–311+G (2d,p) level.

Figure 4 illustrates the percent improvement in the RMS error (red) and maximum absolute error (blue) for GIPAW + MC calculations relative to GIPAW. We compare the percent improvement for the predicted ^17^O *C*_*q*_ values, isotropic shieldings (*σ*_*iso*_) and the CSA tensor elements (*σ*_*ii*_). The RMS error for the ^17^O isotropic chemical shift predictions is 11.53 and 9.35 ppm for GIPAW and GIPAW + MC, respectively. This corresponds to a 19% relative improvement for GIPAW + MC which is in agreement with previous work (Dračínský et al., 2019). However, GIPAW + MC improves the maximum absolute error by only 2%. Similarly, GIPAW + MC improves the RMS error for the ^17^O CSA tensor elements by 4% (19.88 ppm for GIPAW compared to 18.99 ppm for GIPAW + MC). These results are influenced by the larger variation in experimental uncertainties for isotropic shifts and CSA tensor elements (see the Supplementary Material for details). Nevertheless, the roughly two-fold increase in RMS error for CSA tensor elements relative to isotropic shifts is in general agreement with previous results for ^13^C and ^15^N (Hartman et al., 2016; Hartman and Beran, 2018).

Interestingly, the accuracy of GIPAW + MC ^17^O *C*_*q*_ predictions show a larger percent improvement relative to GIPAW compared with both *σ*_*iso*_ and *σ*_*ii*_. In other words, the molecular correction has a more pronounced impact on improving the accuracy of predicted EFG tensors compared to the CS tensor. These results support previous findings which suggest the EFG tensor is a highly local property (Michaelis et al., 2015; Gregorovič, 2020). Unlike the *C*_*q*_ values, the isotropic chemical shift and CSA tensor predictions benefit from a partial correction of systematic error through the application of a regression model discussed in the supporting information. Comparing the percent improvement between the different properties partially accounts for this difference. Nevertheless, care should be exercised when interpreting Figure 4.

### 4.5 Modeling Disorder in the 4-Nitrobenzaldehyde Crystal Structure

NMR parameters derived from the CS and EFG tensors have been used extensively in molecular structure refinement (Olsen et al., 2003; Witter et al., 2006; Harris et al., 2010; Apperley et al., 2012; Harper et al., 2013; Kalakewich et al., 2015; Yang et al., 2016; Soss et al., 2017; Chalek et al., 2021; Wang et al., 2021). Recently, plane-wave EFG tensor predictions involving ^14^N, ^17^O, and ^35^Cl nuclei were used to obtain optimized damping parameters for crystal geometry refinement using Grimme’s DFT-D2 scheme (Holmes and Schurko, 2018). These findings suggest that high-accuracy NMR calculations can be used to help design geometry optimization protocols. Here we examine the sensitivity of ^17^O NMR parameters to subtle changes in geometry using the disordered crystal structure of 4-Nitrobenzaldehyde.

**FIGURE 5:**
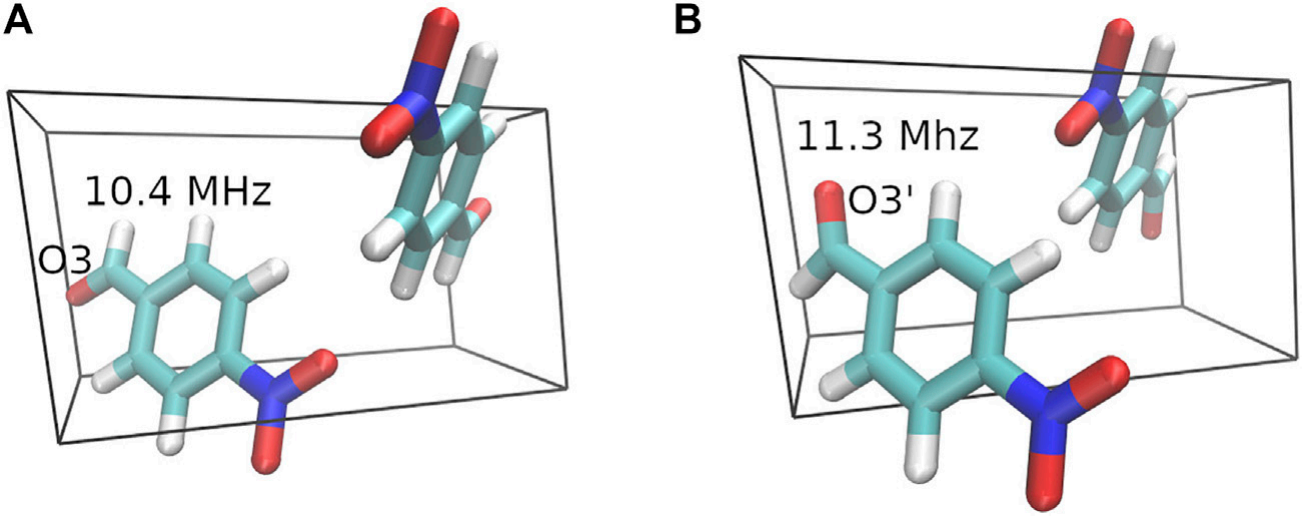
Crystal structures for the two 4-Nitrobenzaldehyde geometries. The dominant crystal structure **(A)** with a 90% occupancy is shown on the left and structure **(B)** with a 10% occupancy is shown on the right. The ^17^O *C*_*q*_ predictions obtained from GIPAW + MC calculations with a PBE0/6-311+G (2d,p) molecular correction are included in the figure.

Disorder is reported in the O3 position for the crystal structure of 4-Nitrobenzaldehyde. The two diffraction structures differ by a 180-degree rotation around the bond between the ring and carbonyl carbon. Figure 5 depicts the unit cell for both crystal structures along with the GIPAW + MC ^17^O *C*_*q*_ predictions. Experimental evidence suggests structure A in Figure 5 is the dominant form, with a 90% occupancy for O_3_. The oxygen labeled O_3_’ in structure B has a 10% occupancy (Jackisch et al., 1989). Table 3 provides the experimental ^17^O *C*_*q*_ value (Wu et al., 2008) and the predicted *C*_*q*_ values for both structures obtained using GIPAW and GIPAW + MC calculations. The final column in Table 3 provides the weighted average of the predicted *C*_*q*_ values based on the experimental occupancy.

**TABLE 3:** Experimental and predicted^17^O *C*_*q*_ values for 4-Nitrobenzaldehyde in MHz. GIPAW + MC results obtained using a PBE0/6-311+G (2d,p) correction.

	Struc. A	Struc. B	Avg.
GIPAW	10.9	11.0	10.9
GIPAW + MC	10.4	11.3	10.5
Exp.			10.7 (2)

The predicted ^17^O quadrupole couplings for structure A from both GIPAW and GIPAW + MC are closer to the experimental value of 10.7 MHz, which agrees with the experimentally derived occupancies. The weighted average *C*_*q*_ predictions for both methods reproduce the experimental value to within the experimental uncertainty (0.2 MHz). However, the GIPAW *C*_*q*_ predictions in Table 3 show that both structures overshoot the experimental value. On the other hand, GIPAW + MC provides *C*_*q*_ predictions which bracket the experimental value. This results in a weighted average of the GIPAW + MC predicted *C*_*q*_ values which more closely matches experiment relative to either structure examined in isolation.

## 5 Conclusion

In summary, the present work establishes the GIPAW + MC method as a simple yet powerful approach for improving the accuracy of traditional plane-wave EFG tensor calculations. Introducing a correction based on the EFG tensor computed on an isolated monomer using a hybrid density functional substantially improves the accuracy of GIPAW calculations. The molecular correction is relatively insensitive to the choice of basis set, ensuring the cost of the molecular correction is a small fraction of the corresponding GIPAW calculation. In addition to improving the accuracy of predicted *C*_*q*_ values, we have shown the molecular correction improves the accuracy of predicted ^17^O CSA tensor elements. Comparing the relative improvement obtained through introducing a molecular correction to both EFG and CS tensor predictions we find a larger improvement in the accuracy of predicted EFG tensors. Finally, we apply GIPAW + MC EFG tensor calculations to the disordered crystal structure of 4-Nitrobenzaldehyde and show the molecular correction improves resolution between the different crystal geometries present in the disordered crystal (Stoychev et al., 2018).

## Data Availability

The original contributions presented in the study are included in the article/Supplementary Material, further inquiries can be directed to the corresponding author.
