# Supplementary figures and images for: Fast and Accurate Electric Field Gradient Calculations in Molecular Solids With Density Functional Theory
Source: Front Chem. 2021 Oct 7;9:751711. doi: 10.3389/fchem.2021.751711 (PMC8529703; doi:10.3389/fchem.2021.751711)

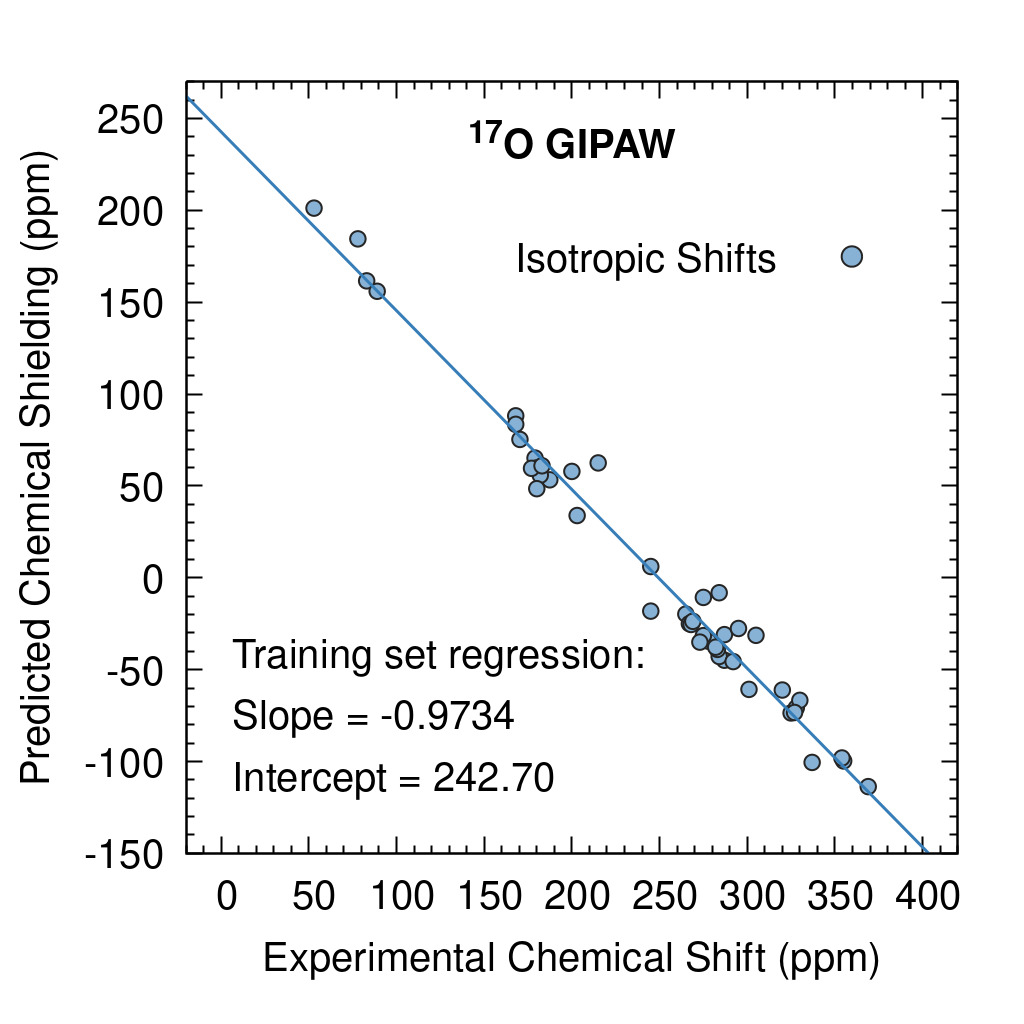

Supplement: Supplementary file 1 [file Image3.JPEG]

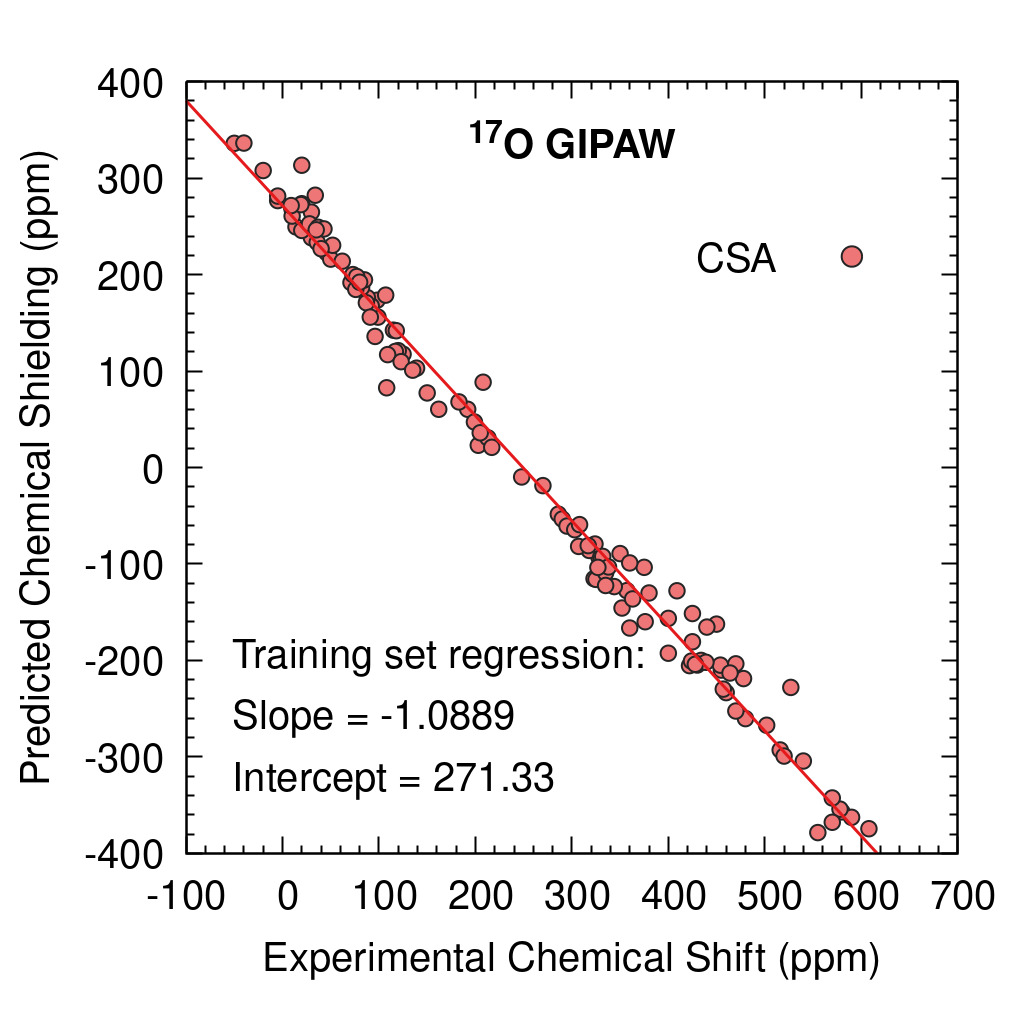

Supplement: Supplementary file 2 [file Image1.JPEG]

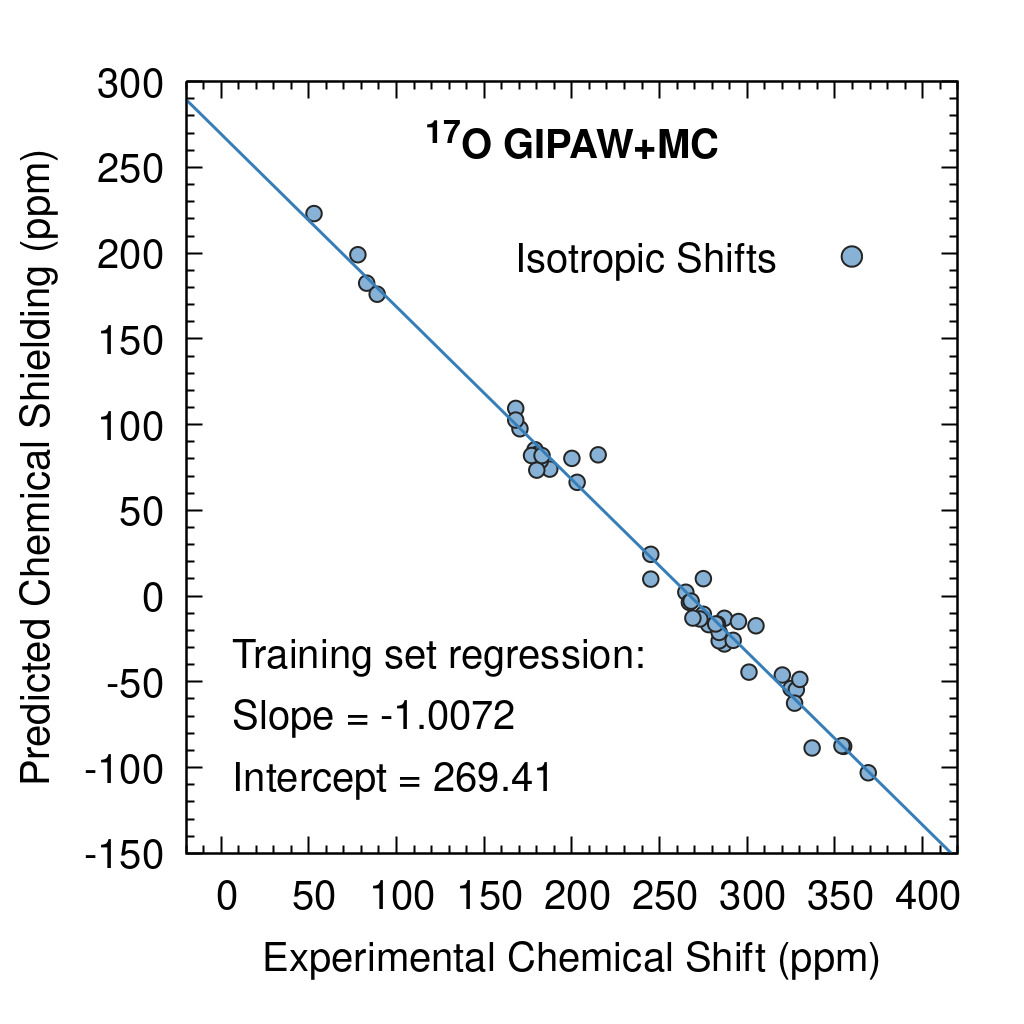

Supplement: Supplementary file 3 [file Image4.JPEG]

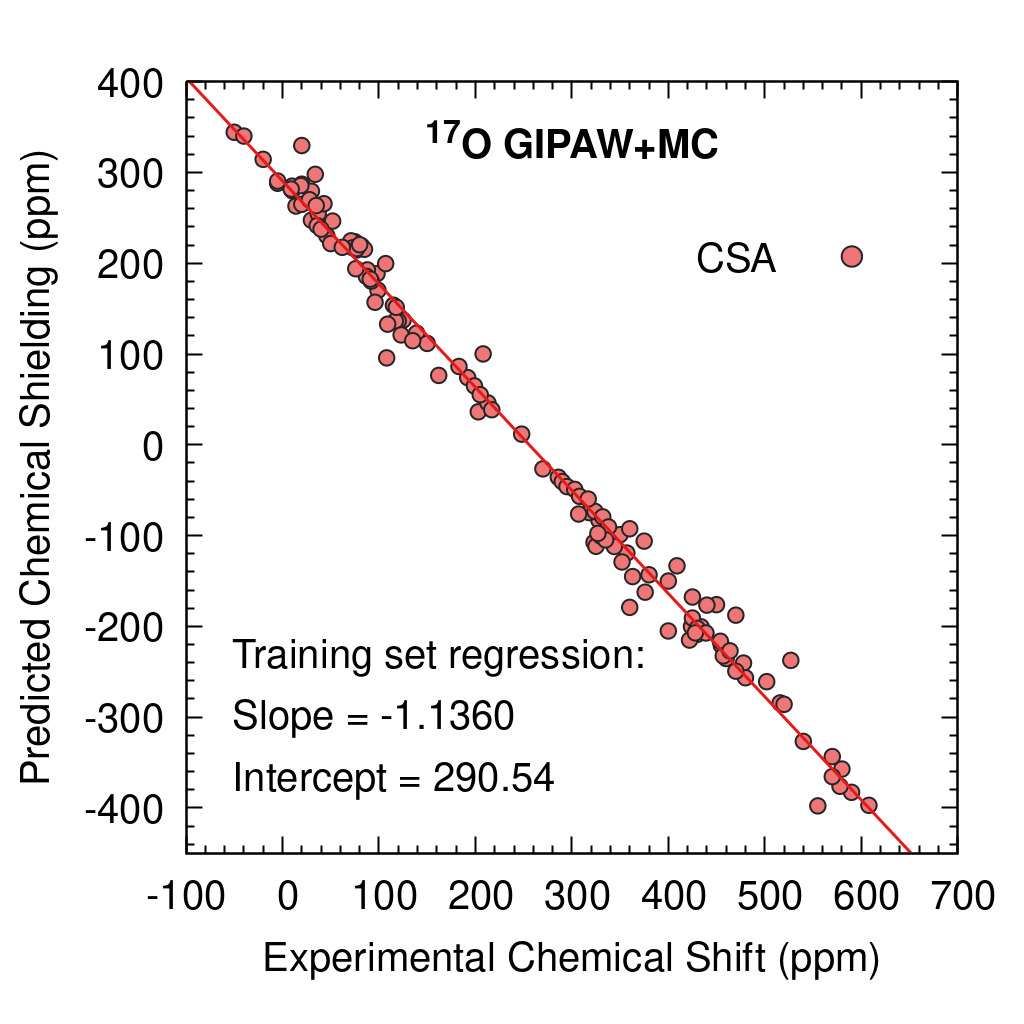

Supplement: Supplementary file 4 [file Image2.JPEG]
