# Supplementary material for: Fast and Accurate Electric Field Gradient Calculations in Molecular Solids With Density Functional Theory
Source: Front Chem. 2021 Oct 7;9:751711. doi: 10.3389/fchem.2021.751711 (PMC8529703; doi:10.3389/fchem.2021.751711)
Supplement: Supplementary file 5 [file DataSheet1.pdf]

Supplementary Material

1 SUPPLEMENTARY DATA

This supporting information provides plots and the corresponding regression parameters for the four different regression models used to map predicted chemical shifts and principal components to experiment. It includes complete tables of the experimental, GIPAW, and GIPAW+MC chemical shifts. It also provides an comparison of the accuracy of predicted ^{17}O C_q values obtained from fixed-cell and fixed-volume geometry optimization protocols. Finally, a detailed table is provided which includes 2-D structures for all species included in the ^{17}O benchmark set. CIF files containing the optimized geometries of all crystals in the benchmark set are provided separately.

2 LINEAR REGRESSION MODELS

The experimentally observed chemical shift δ_i represents the difference between the absolute chemical shielding σ_i of nucleus i and the absolute shielding of a reference compound (σ_{ref}). Liquid water is used as the reference compound for the ^{17}O data. Therefore, comparing predicted shifts with experiment requires mapping between the computed absolute shieldings σ_i and the experimentally referenced chemical shift data. We perform the mapping using a linear regression approach which simultaneously performs shift referencing and partially corrects for systematic errors in the calculations.

$$\delta_i = A\sigma_i + B \tag{S1}$$

In the absence of systematic error, A would take a value of -1, and B would represent the absolute shielding of the reference compound. We obtain both parameters using a least-squares fit between the experimental and calculated values for each computational method. Figure S1 illustrates the application of this approach to the ^{17}O benchmark set for both the isotropic shifts and the principal components.

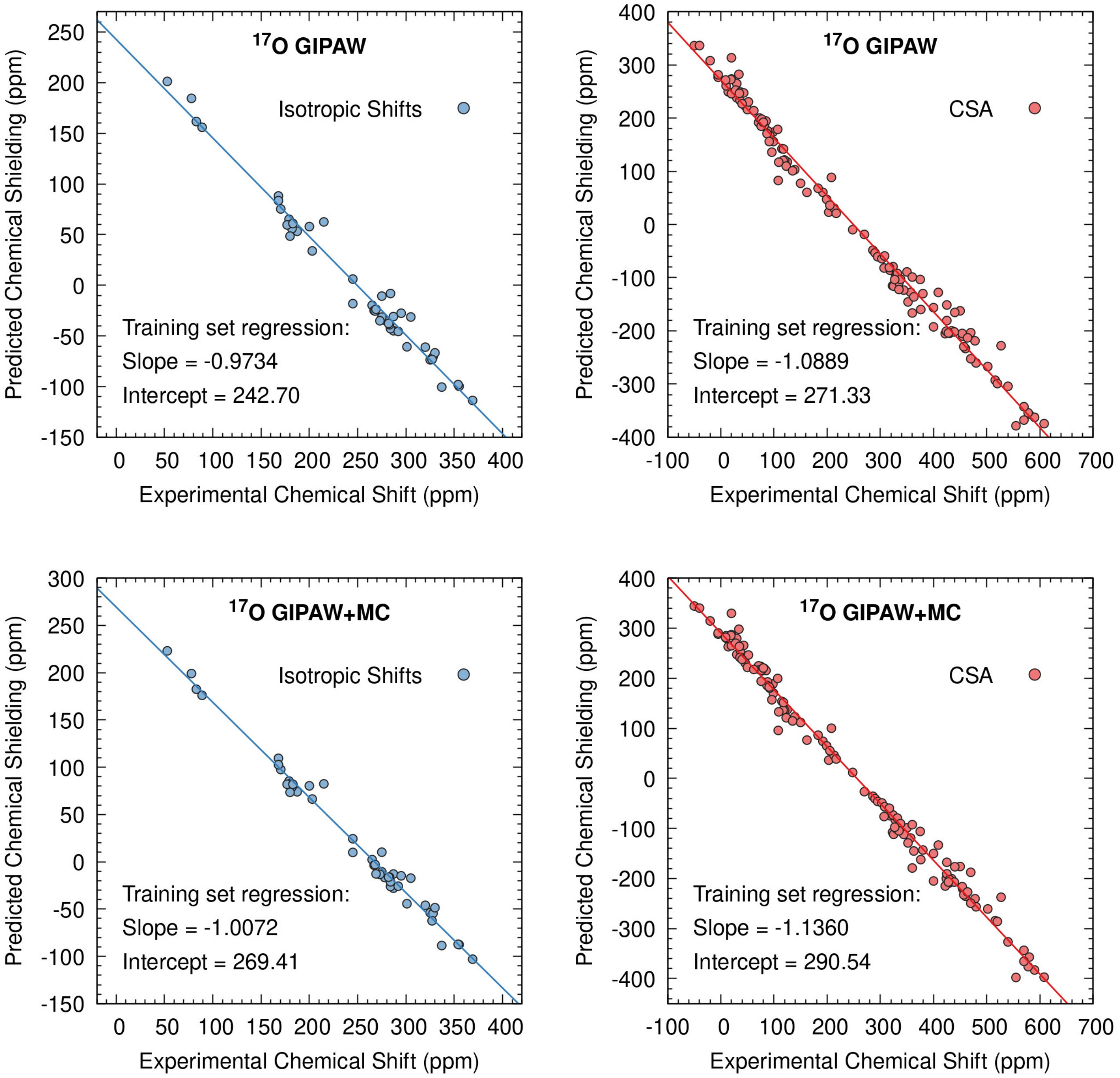

Figure S1. Plot of calculated vs. experimental ^{17}O CS tensor data for the GIPAW isotropic shifts, and principal components (top figures), and the GIPAW+MC isotropic shifts and principal components (bottom figures). The GIPAW calculations were carried out as described in the main text using the PBE density functional, and the GIPAW+MC calculations were performed using a PBE0/6-311+G(2d,p) molecular correction. The individual regression parameters are provided for each model.

3 TABULATED EXPERIMENTAL AND PREDICTED CHEMICAL SHIELDING TENSOR DATA

Tables S1 and S2 list the experimental and predicted CS tensor data for every species in the ^{17}O benchmark set with available experimental data. Experimental isotropic ^{17}O chemical shift data was available for 22 crystal structures providing a total of 46 individual isotropic shifts (Table S1) and 138 principal components (Table S2).

Table S1: Experimental ^{17}O isotropic chemical shift values with the reported uncertainty for each structure in the benchmark set. Calculated δ_{iso} for GIPAW and GIPAW+MC calculations provided along with the absolute errors. All values are reported in ppm and the GIPAW+MC calculations were performed with a PBE0/6-311+G(2d,p) molecular correction. The absolute shieldings can be obtained from the scaled values using the regression model provided in Figure S1.

				GIPAW		GIPAW+MC	
CSD Code	Atom	Exp. δ_{iso}	(δ_{iso})	Calc. δ_{iso}	Abs. Error	Calc. δ_{iso}	Abs. Error
TAURIN01	O1	170.4	0.2	171.98	-1.58	170.58	-0.18
	O2	179.0	0.2	182.40	-3.40	182.70	-3.70
	O3	187.4	0.2	194.55	-7.15	193.96	-6.56
BZANIL02	O1	320.0	5	312.07	7.93	313.15	6.85
TPEPHO02	O1	53.0	0.2	42.65	10.35	45.99	7.01
PHALNC01	O1	355.0	2	351.76	3.24	354.46	0.54
	O2	180.0	2	187.90	-7.90	185.45	-5.45
VALEHC11	O1	354.0	2	350.09	3.91	354.09	-0.09
	O2	182.0	2	191.99	-9.99	189.11	-7.11
GLUTAM01	O1	301.0	1	311.73	-10.73	311.48	-10.48
	O2	265.0	1	269.63	-4.63	265.22	-0.22
LHISTD13	O1	278.0	1	284.84	-6.84	283.98	-5.98
	O2	275.0	1	281.66	-6.66	277.99	-2.99
THYMIN01	O1	200.0	5	189.86	10.14	187.71	12.29
	O2	325.0	5	324.91	0.09	320.97	4.03
CUWKIO	O1	305.0	2	281.50	23.50	284.65	20.35
	O2	180.0	2	199.51	-19.51	194.53	-14.53

Continued on next page

Table S1 – *Continued from previous page*

				GIPAW		GIPAW+MC	
CSD Code	Atom	Exp. δ_{iso}	(δ_{iso})	Calc. $delta_{iso}$	Abs. Error	Calc. δ_{iso}	Abs. Error
URACIL	O1	245.0	5	243.02	1.98	243.28	1.72
	O2	275.0	5	260.31	14.69	257.37	17.63
MOHCIW	O1	337.0	2	352.57	-15.57	355.39	-18.39
	O2	168.0	2	158.78	9.22	158.79	9.21
LALNIN12	O1	287.0	1	295.44	-8.44	295.16	-8.16
	O2	267.0	1	274.95	-7.95	271.12	-4.12
ALAHCL	O1	328.0	1	322.04	5.96	321.62	6.38
	O2	177.0	2	188.13	-11.13	186.06	-9.06
MBNZAM10	O1	287.0	5	281.12	5.89	280.29	6.71
ACANIL03	O1	330.0	5	317.83	12.17	315.66	14.34
LTHREO01	O1	284.0	2	293.32	-9.32	293.34	-9.34
	O2	283.0	2	289.37	-6.37	283.62	-0.62
LTYROS11	O1	292.0	1	296.17	-4.17	293.07	-1.07
	O2	268.0	1	275.45	-7.45	270.40	-2.40
LTYRHC10	O1	327.0	0.5	324.63	2.37	329.44	-2.44
	O2	183.0	0.5	186.70	-3.70	186.09	-3.09
	O3	83.0	0.5	83.31	-0.31	86.30	-3.30
SALIAC12	O1	168.0	1	163.52	4.48	165.55	2.45
	O2	284.0	1	257.68	26.32	288.49	-4.49
	O3	89.0	1	89.13	-0.13	92.69	-3.69
ACSALA17	O1	273.0	1	285.29	-12.29	280.70	-7.70
	O2	215.0	1	185.10	29.90	185.68	29.32
	O3	203.0	1	214.52	-11.52	201.57	1.43
	O4	369.0	1	366.11	2.89	369.75	-0.75
TICHOC	O1	245.0	2	267.98	-22.98	257.63	-12.63
	O2	295.0	2	277.67	17.33	282.16	12.84
	O3	78.0	2	59.84	18.16	69.82	8.18

Continued on next page

Table S1 – *Continued from previous page*

				GIPAW		GIPAW+MC	
CSD Code	Atom	Exp. δ_{iso}	(δ_{iso})	Calc. $delta_{iso}$	Abs. Error	Calc. δ_{iso}	Abs. Error
LASPRT	O1	282.0	1	288.11	-6.11	283.59	-1.59
	O2	269.0	1	273.66	-4.66	280.17	-11.17
RMSE					11.46		9.30
$\|\|$Max Error$\|\|$					29.90		29.32

Table S2: Experimental ^{17}O principal components with the reported uncertainty for each structure in the benchmark set. Calculated principal components for GIPAW and GIPAW+MC calculations provided along with the absolute errors. All values are reported in ppm and the GIPAW+MC calculations were performed with a PBE0/6-311+G(2d,p) molecular correction. The absolute principal components can be obtained from the scaled values using the regression model provided in Figure S1.

					GIPAW		GIPAW+MC	
CSD Code	Atom	Atom	Exp. δ_{iso}	(δ_{iso})	Calc. δ_{iso}	Abs. Error	Calc. δ_{iso}	Abs. Error
TAURIN01	O1	σ_{11}	203.0	10	228.00	-25.00	223.76	-20.76
		σ_{22}	192.0	10	193.72	-1.72	190.54	1.46
		σ_{33}	115.0	10	118.43	-3.43	120.40	-5.40
	O2	σ_{11}	213.0	10	221.02	-8.02	215.14	-2.14
		σ_{22}	199.0	10	205.81	-6.81	198.66	0.34
		σ_{33}	125.0	10	141.26	-16.26	134.89	-9.89
	O3	σ_{11}	217.0	10	229.98	-12.98	221.72	-4.72
		σ_{22}	205.0	10	216.10	-11.10	207.14	-2.14
		σ_{33}	139.0	10	154.58	-15.58	147.51	-8.51
BZANIL02	O1	σ_{11}	580.0	5	577.04	2.96	570.09	9.91
		σ_{22}	450.0	5	398.29	51.71	410.72	39.28
		σ_{33}	-50.0	5	-59.46	9.46	-47.27	-2.73
TPEPHO02	O1	σ_{11}	150.0	–	178.15	-28.15	157.45	-7.45
		σ_{22}	14.0	–	20.08	-6.08	24.22	-10.22
		σ_{33}	-5.0	–	-4.94	-0.06	2.16	-7.16
PHALNC01	O1	σ_{11}	590.0	9	582.22	7.78	592.68	-2.68
		σ_{22}	455.0	9	442.04	12.96	450.24	4.76
		σ_{33}	20.0	9	-1.96	21.96	3.04	16.96
	O2	σ_{11}	323.0	9	354.95	-31.95	350.37	-27.37

Continued on next page

Table S2 – *Continued from previous page*

					GIPAW		GIPAW+MC	
CSD Code	Atom	Atom	Exp. δ_{iso}	(δ_{iso})	Calc. δ_{iso}	Abs. Error	Calc. δ_{iso}	Abs. Error
		σ_{22}	120.0	9	137.95	-17.95	135.72	-15.72
		σ_{33}	98.0	9	89.93	8.07	89.75	8.25
VALEHC11	O1	σ_{11}	578.0	9	574.44	3.56	586.82	-8.82
		σ_{22}	454.0	9	437.33	16.67	446.22	7.78
		σ_{33}	30.0	9	6.04	23.96	9.88	20.12
	O2	σ_{11}	330.0	9	349.11	-19.11	346.25	-16.25
		σ_{22}	117.0	9	138.70	-21.70	135.72	-18.72
		σ_{33}	99.0	9	106.00	-7.00	105.93	-6.93
GLUTAM01	O1	σ_{11}	516.0	4	518.09	-2.09	505.98	10.02
		σ_{22}	357.0	4	366.39	-9.39	360.67	-3.67
		σ_{33}	30.0	4	30.47	-0.47	37.95	-7.95
	O2	σ_{11}	434.0	4	432.86	1.14	432.22	1.78
		σ_{22}	286.0	4	293.59	-7.59	287.23	-1.23
		σ_{33}	75.0	4	75.59	-0.59	58.86	16.14
LHISTD13	O1	σ_{11}	460.0	4	463.52	-3.52	462.94	-2.94
		σ_{22}	328.0	4	334.47	-6.47	328.74	-0.74
		σ_{33}	46.0	4	44.84	1.16	53.09	-7.09
	O2	σ_{11}	430.0	4	437.21	-7.21	439.55	-9.55
		σ_{22}	318.0	4	328.10	-10.10	321.45	-3.45
		σ_{33}	77.0	4	68.99	8.01	66.78	10.22
THYMIN01	O1	σ_{11}	290.0	10	298.22	-8.22	291.50	-1.50
		σ_{22}	270.0	10	266.43	3.57	279.07	-9.07
		σ_{33}	20.0	10	23.45	-3.45	22.53	-2.53
	O2	σ_{11}	570.0	10	586.93	-16.93	577.47	-7.47
		σ_{22}	360.0	10	402.00	-42.00	413.35	-53.35
		σ_{33}	20.0	10	-38.64	58.64	-34.43	54.43
CUWKIO	O1	σ_{11}	478.0	9	450.13	27.87	467.34	10.66
		σ_{22}	409.0	9	366.51	42.49	373.09	35.91
		σ_{33}	28.0	9	17.22	10.78	18.10	9.90
	O2	σ_{11}	325.0	9	355.67	-30.67	354.19	-29.19
		σ_{22}	108.0	9	173.25	-65.25	171.45	-63.45
		σ_{33}	107.0	9	85.07	21.93	80.08	26.92
URACIL	O1	σ_{11}	400.0	10	392.82	7.18	387.85	12.15
		σ_{22}	330.0	10	334.35	-4.35	344.20	-14.20
		σ_{33}	10.0	10	3.50	6.50	4.99	5.01

Continued on next page

Table S2 – *Continued from previous page*

					GIPAW		GIPAW+MC	
CSD Code	Atom	Atom	Exp. δ_{iso}	(δ_{iso})	Calc. δ_{iso}	Abs. Error	Calc. δ_{iso}	Abs. Error
	O2	σ_{11}	470.0	10	436.10	33.90	420.93	49.07
		σ_{22}	350.0	10	331.13	18.87	342.88	7.12
		σ_{33}	10.0	10	9.80	0.20	9.26	0.74
MOHCIW	O1	σ_{11}	555.0	9	596.73	-41.73	605.82	-50.82
		σ_{22}	422.0	9	437.80	-15.80	445.04	-23.04
		σ_{33}	34.0	9	-10.06	44.06	-6.51	40.51
	O2	σ_{11}	324.0	9	322.03	1.97	320.26	3.74
		σ_{22}	92.0	9	95.25	-3.25	97.01	-5.01
		σ_{33}	88.0	9	87.46	0.54	86.19	1.81
LALNIN12	O1	σ_{11}	480.0	5	488.27	-8.27	481.55	-1.55
		σ_{22}	344.0	5	362.55	-18.55	354.37	-10.37
		σ_{33}	37.0	5	20.43	16.57	31.59	5.41
	O2	σ_{11}	424.0	5	433.48	-9.48	431.90	-7.90
		σ_{22}	295.0	5	304.92	-9.92	296.27	-1.27
		σ_{33}	82.0	5	77.91	4.09	62.93	19.07
ALAHCL	O1	σ_{11}	540.0	5	528.64	11.36	543.33	-3.33
		σ_{22}	425.0	5	415.08	9.92	423.67	1.33
		σ_{33}	19.0	5	-1.12	20.12	4.46	14.54
	O2	σ_{11}	335.0	5	349.44	-14.44	345.74	-10.74
		σ_{22}	109.0	5	141.68	-32.68	138.77	-29.77
		σ_{33}	87.0	5	92.32	-5.32	92.39	-5.39
MBNZAM10	O1	σ_{11}	520.0	5	523.92	-3.92	507.37	12.63
		σ_{22}	380.0	5	368.63	11.37	381.73	-1.73
		σ_{33}	-40.0	5	-59.71	19.71	-43.63	3.63
ACANIL03	O1	σ_{11}	570.0	5	563.80	6.20	558.02	11.98
		σ_{22}	440.0	5	401.09	38.91	411.15	28.85
		σ_{33}	-20.0	5	-33.57	13.57	-21.08	1.08
LTHREO01	O1	σ_{11}	470.0	6	481.01	-11.01	475.22	-5.22
		σ_{22}	332.0	6	333.77	-1.77	325.61	6.39
		σ_{33}	50.0	6	50.80	-0.80	60.60	-10.60
	O2	σ_{11}	457.0	6	460.15	-3.15	460.45	-3.45
		σ_{22}	307.0	6	324.29	-17.29	322.85	-15.85
		σ_{33}	85.0	6	70.53	14.47	66.13	18.87
LTYROS11	O1	σ_{11}	502.0	4	494.53	7.47	485.39	16.61

Continued on next page

Table S2 – *Continued from previous page*

					GIPAW		GIPAW+MC	
CSD Code	Atom	Atom	Exp. δ_{iso}	(δ_{iso})	Calc. δ_{iso}	Abs. Error	Calc. δ_{iso}	Abs. Error
		σ_{22}	338.0	4	343.89	-5.89	335.37	2.63
		σ_{33}	36.0	4	34.79	1.21	43.24	-7.24
	O2	σ_{11}	430.0	4	436.13	-6.13	433.70	-3.70
		σ_{22}	303.0	4	308.39	-5.39	298.95	4.05
		σ_{33}	71.0	4	73.11	-2.11	57.95	13.05
SALIAC12	O1	σ_{11}	308.0	10	303.55	4.45	305.67	2.33
		σ_{22}	123.0	10	148.39	-25.39	149.19	-26.19
		σ_{33}	73.0	10	65.51	7.49	64.72	8.28
	O2	σ_{11}	425.0	10	388.18	36.82	403.30	21.70
		σ_{22}	375.0	10	344.20	30.80	349.13	25.87
		σ_{33}	52.0	10	37.60	14.40	38.81	13.19
	O3	σ_{11}	162.0	10	193.75	-31.75	188.47	-26.47
		σ_{22}	96.0	10	124.39	-28.39	117.52	-21.52
		σ_{33}	9.0	10	-0.20	9.20	7.80	1.20
ACSALA17	O1	σ_{11}	400.0	10	425.99	-25.99	436.25	-36.25
		σ_{22}	376.0	10	396.00	-20.00	398.63	-22.63
		σ_{33}	43.0	10	22.05	20.95	21.99	21.01
	O2	σ_{11}	360.0	10	339.99	20.01	337.14	22.86
		σ_{22}	208.0	10	167.96	40.04	167.62	40.38
		σ_{33}	77.0	10	67.38	9.62	65.47	11.53
	O3	σ_{11}	335.0	10	361.50	-26.50	347.89	-12.89
		σ_{22}	183.0	10	186.74	-3.74	179.78	3.22
		σ_{33}	91.0	10	106.00	-15.00	95.09	-4.09
	O4	σ_{11}	608.0	10	593.06	14.94	605.26	2.74
		σ_{22}	464.0	10	444.84	19.16	455.55	8.45
		σ_{33}	35.0	10	22.88	12.12	23.78	11.22
TICHOC	O1	σ_{11}	352.0	10	382.95	-30.95	369.32	-17.32
		σ_{22}	248.0	10	258.17	-10.17	245.35	2.65
		σ_{33}	135.0	10	156.49	-21.49	154.81	-19.81
	O2	σ_{11}	527.0	10	458.55	68.45	464.76	62.24
		σ_{22}	363.0	10	374.29	-11.29	383.47	-20.47
		σ_{33}	-5.0	10	-9.24	4.24	0.02	-5.02
	O3	σ_{11}	118.0	10	118.93	-0.93	122.44	-4.44
		σ_{22}	76.0	10	79.58	-3.58	85.01	-9.01
		σ_{33}	40.0	10	40.87	-0.87	46.67	-6.67
LASPRT	O1	σ_{11}	439.0	4	434.60	4.40	438.18	0.83
		σ_{22}	327.0	4	344.25	-17.25	341.68	-14.68

Continued on next page

Table S2 – *Continued from previous page*

CSD Code	Atom	Atom	Exp. δ_{iso}	(δ_{iso})	GIPAW Calc. δ_{iso}	GIPAW Abs. Error	GIPAW+MC Calc. δ_{iso}	GIPAW+MC Abs. Error
		σ_{33}	80.0	4	72.76	7.24	61.72	18.28
	O2	σ_{11}	428.0	4	436.52	-8.52	438.10	-10.10
		σ_{22}	317.0	4	323.56	-6.56	308.33	8.67
		σ_{33}	62.0	4	52.77	9.23	64.25	-2.25
RMSE						20.12		18.66
$\|\|$Max Error$\|\|$						68.45		63.45

4 EFFECT OF FIXED-VOLUME GEOMETRY OPTIMIZATION ON THE ACCURACY OF PREDICTED ^{17}O C_Q VALUES.

Experimental crystal structures are typically subjected to all-atom geometry optimizations using fixed lattice parameters. This approach has proven highly successful in modeling room temperature crystal structures. However, using fixed lattice parameters could introduce anisotropic strain resulting in errors in the optimized geometries. To explore this possibility, we performed all-atom fixed-volume optimizations and compared the accuracy of the predicted ^{17}O C_q values with those obtained from the all-atom fixed-cell optimizations used throughout this work. The fixed-volume geometry optimizations were performed using the same protocol outlined in Section 3.1 with a fixed-volume constraint applied in place of the fixed-cell constraint.

Figure S2 illustrates the error distributions for predicted ^{17}O C_q values obtained from fixed-cell optimizations (purple and red) and fixed-volume optimizations (green). In general, fixed-volume geometry optimizations result in minor changes to the crystal structure in our test set. In some cases, the fixed-volume optimizations had a negligible impact on the geometry. For example, fixed-volume optimization of the N-Methylbenzamide crystal (MBNZAM10) resulted in the same crystal structure obtained from the fixed-cell optimization. The similar structures obtained from the two optimization protocols result in similar error distributions. However, structural changes at certain atom sites resulted in significantly larger errors in the predicted C_q values. The large errors result in much longer whiskers in Figure S2 with a $\sim$75% increase in the maximum error. These findings suggest the fixed-cell optimization protocol provides more accurate room temperature crystal structures.

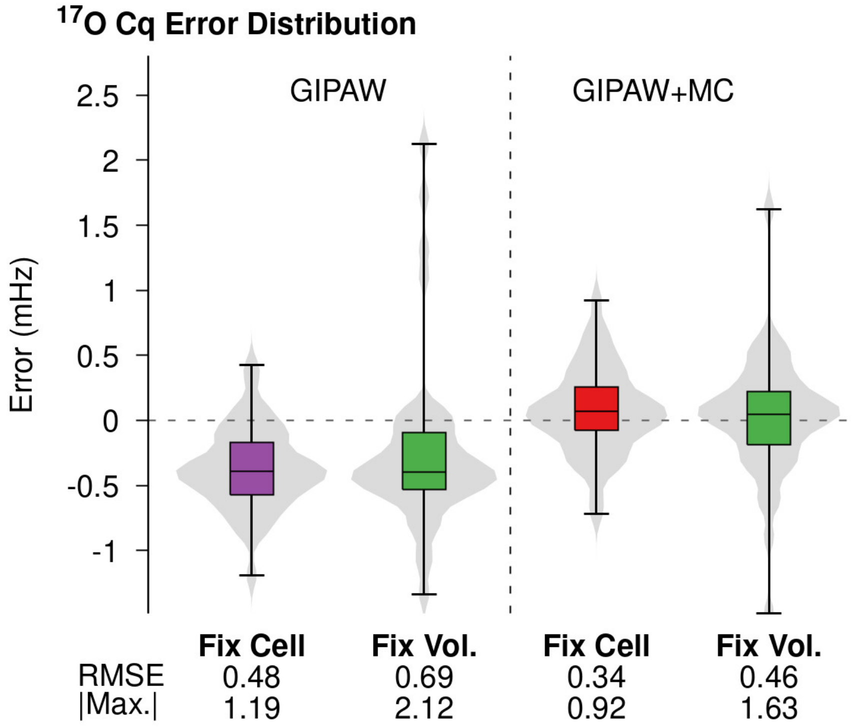

Figure S2. Error distributions for the predicted ^{17}O C_q values obtained from GIPAW (left) and GIPAW+MC (right) calculations. The error distributions corresponding to all-atom fixed cell optimized geometries are shown in purple and red for GIPAW and GIPAW+MC calculations, respectively. The error distributions obtained from all-atom fixed volume geometry optimizations are shown in green. The molecular correction for the GIPAW+MC calculations were performed at the PBE0/6-311+G(2d,p) level.

5 CRYSTAL STRUCTURES

Table S3: Crystal structures included in the ^{17}O benchmark set.

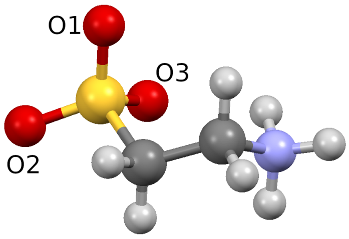

TAURIN01

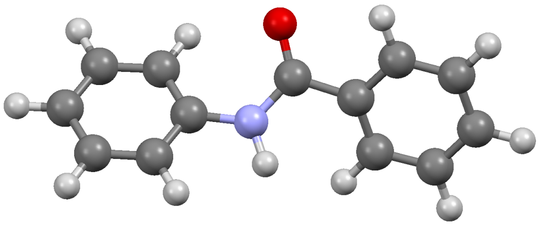

BZANIL02

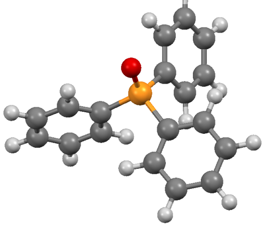

TPEPHO02

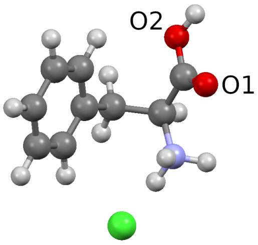

PHALNC01

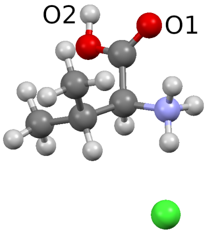

VALEHC11

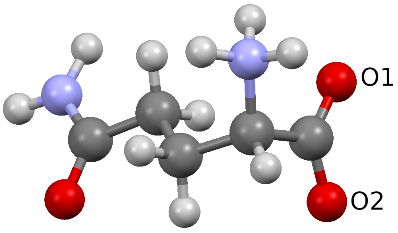

GLUTAM01

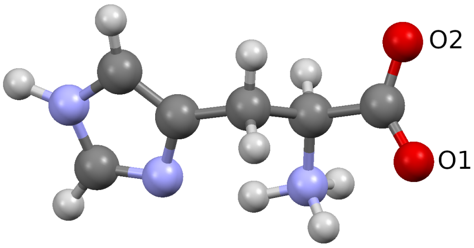

LHISTD13

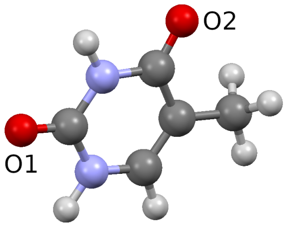

THYMIN01

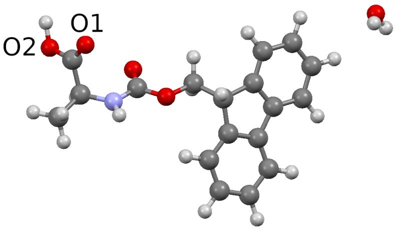

CUWKIO

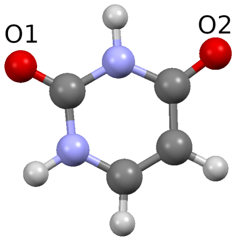

URACIL

Table S3 – *Continued ^{17}O benchmark set from previous page*

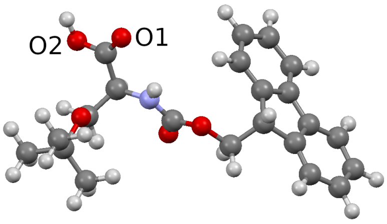

MOHCIW

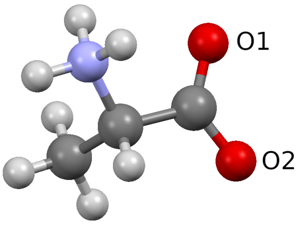

LALNIN12

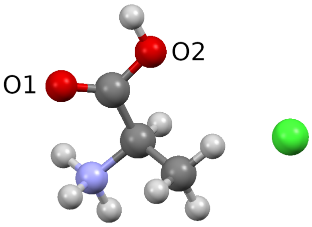

ALAHCL

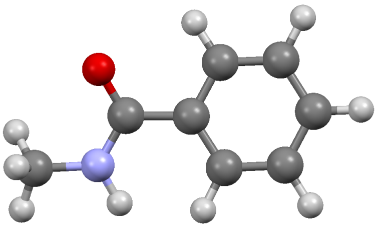

MBNZAM10

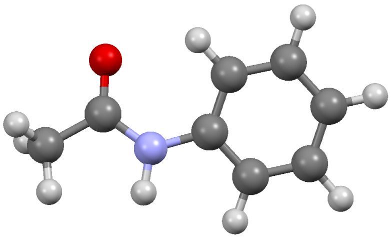

ACANIL03

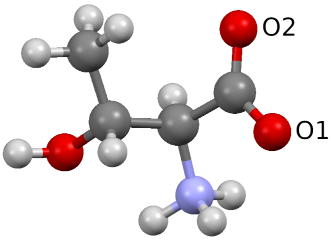

LTHREO01

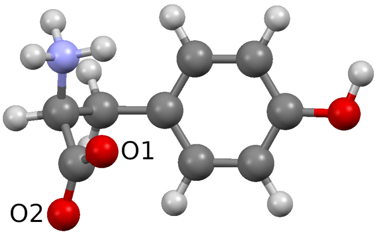

LTYROS11

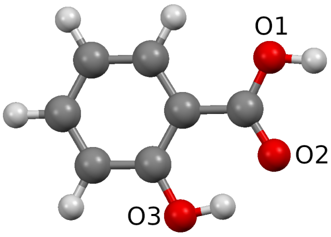

SALIAC12

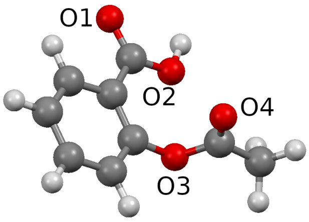

ACSALA17

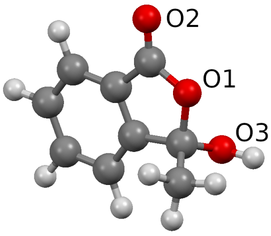

TICHOC

Table S3 – *Continued ^{17}O benchmark set from previous page*

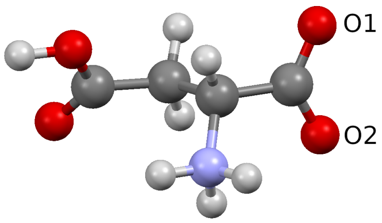

LASPRT

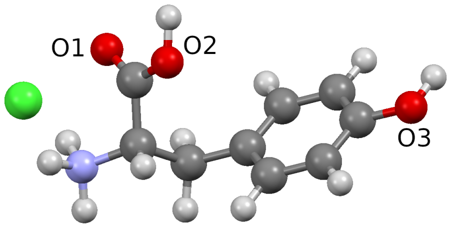

LTYRHC10
